# Endoglucanase activity at a second site in *Pyrococcus furiosus* triosephosphate isomerase—Promiscuity or compensation for a metabolic handicap?

**DOI:** 10.1002/2211-5463.12249

**Published:** 2017-07-11

**Authors:** Prerna Sharma, Purnananda Guptasarma

**Affiliations:** ^1^ Department of Biological Sciences Centre for Protein Science Design and Engineering (CPSDE) Indian Institute of Science Education and Research (IISER) Mohali Punjab; ^2^ Division of Protein Science and Engineering CSIR‐ Institute of Microbial Technology Chandigarh India

**Keywords:** enzyme inhibitors, enzyme kinetics, enzyme mutations, enzyme promiscuity, glycolysis metabolism, triosephosphate isomerase

## Abstract

The eight‐stranded (β/α)_8_ barrel fold known as the Triosephosphate isomerase (TIM) barrel is the most commonly observed fold in enzymes, displaying an eightfold structural symmetry. The sequences and structures of different TIM barrel enzymes suggest that nature exploits the modularity inherent in the eightfold symmetry to generate enzymes with diverse enzymatic activities and, in certain cases, more than one catalytic activity per enzyme. Here, we report the discovery, verification, and characterization of such an additional activity, a novel endoglucanase/cellulase activity in what is otherwise a triosephosphate isomerase from the hyperthermophile archaeon *Pyrococcus furiosus* (PfuTIM). The activity is seen in two different ranges of temperatures, with one maximum at 40 °C and a second maximum close to 100 °C. The endoglucanase/cellulase activity is inhibited by norharman, a TIM inhibitor, which is suspected to bind at a site different to that of the regular substrate, glyceraldehyde‐3‐phosphate (G3P). However, endoglucanase/cellulose activity is not inhibited either by G3P analogs or by glycine‐scanning mutations involving residues in loops 1, 4, and 6 of PfuTIM, which are known to be important for TIM activity. It appears, therefore, that two different sites on PfuTIM are responsible for the observed TIM and endoglucanase activities. We discuss possible correlations between this discovery and certain unusual features of the glycolytic pathway in *P. furiosus*.

**Enzyme:**

*Pyrococcus furiosus* Triosephosphate isomerase (EC:5.3.1.1)

**Abbreviations**
3‐PPA: 3‐phosphopropanoic acid
Bgl: beta‐glucosidase
EglA: endoglucanase A
EMP: Embden–Meyerhof–Parnas pathway
G3P: glyceraldehyde‐3‐phosphate
ITC: isothermal titration calorimetry
LamA: laminarinase A
Pfu: Pyrococcus furiosus
TIM/TPI: triosephosphate isomerase

This study describes an accidental discovery and its investigation and post facto rationalization. Briefly, during some studies on cellulases being engineered to form ‘chimeras’, we have to use as a control an enzyme, which was expected to display no detectable endoglucanase/cellulase activity of its own, deployed only to provide ‘baseline’ data for comparisons with any activity detected in the chimeric cellulases. The control enzyme was PfuTIM, a triosephosphate isomerase (TIM) from the glycolytic pathway of the hyperthermophile archaeon, *Pyrococcus furiosus*. We had used PfuTIM, a well‐studied protein/enzyme from our own laboratory merely because it was available. To our considerable surprise, we discovered that PfuTIM displays substantial endoglucanase/cellulase activity.

Following the elimination of all other possibilities, and after confirming beyond reasonable doubt that the additional activity was indeed associated with PfuTIM, we considered the possibility that PfuTIM is an enzyme with two activities: its well‐known enzymatic activity as a triosephosphate isomerase, and an additional hitherto‐unsuspected endoglucanase/cellulase activity. We wondered whether PfuTIM had developed what is called ‘enzymatic promiscuity’, during its evolution within the genome of *P. furiosus*.

In principle, when an enzyme acquires a novel activity during evolution, both original and new activities can coexist and simultaneously persist, especially if both activities appear to serve the host microorganism suitably and remain somehow ‘selected for’ during the organism's growth and multiplication, and further evolution. Also, in principle, an additional activity can potentially involve the very same region of the protein known to be responsible for the primary activity (i.e., the two functions can involve common catalytically active residues), or involve altogether different regions of the enzyme. In general, primitive organisms may be considered to be more likely to host proteins with multiple functions, as such functions would have the scope to become differentiated and separated into tasks associated with individual, specialized proteins in more complex organisms with larger genomes as well as proteomes, and larger numbers of enzyme isoforms produced by different sets of duplicated genes.

We describe below the discovery and detailed characterization of PfuTIM's second function as an endoglucanase/cellulase. To the best of our knowledge, this is the first report of an additional activity in a TIM from any organism, although instances are known of additional activities being associated with other enzymes that adopt the TIM barrel type of fold 1, 2. TIM catalyzes the interconversion of dihydroxy acetone phosphate (DHAP) and glyceraldehyde‐3‐phosphate (G3P) during glycolysis. It is highly thermostable, owing to its evolutionary origins in *P. furiosus*, and resistant to denaturation by heat or denaturant, requiring a combination of both heat and denaturant for unfolding 3. We report that PfuTIM shows endoglucanase/cellulase activity in the temperature range of 30–40 °C, as well as at temperatures of 90 °C and above, but not at any intermediate temperatures between these two ranges.

By being capable of playing dual enzymatic roles, PfuTIM could potentially represent an example of divergent evolution as well as an example of a survival strategy for an organism with a possible metabolic handicap, in which a TIM from the glycolytic pathway doubles up as a generator of at least some of the glucose that feeds into the pathway.

## Results

### Observations of cellulase activity in PfuTIM

#### Miller's DNSA stopping‐based cellulase assay

**Figure 1:**
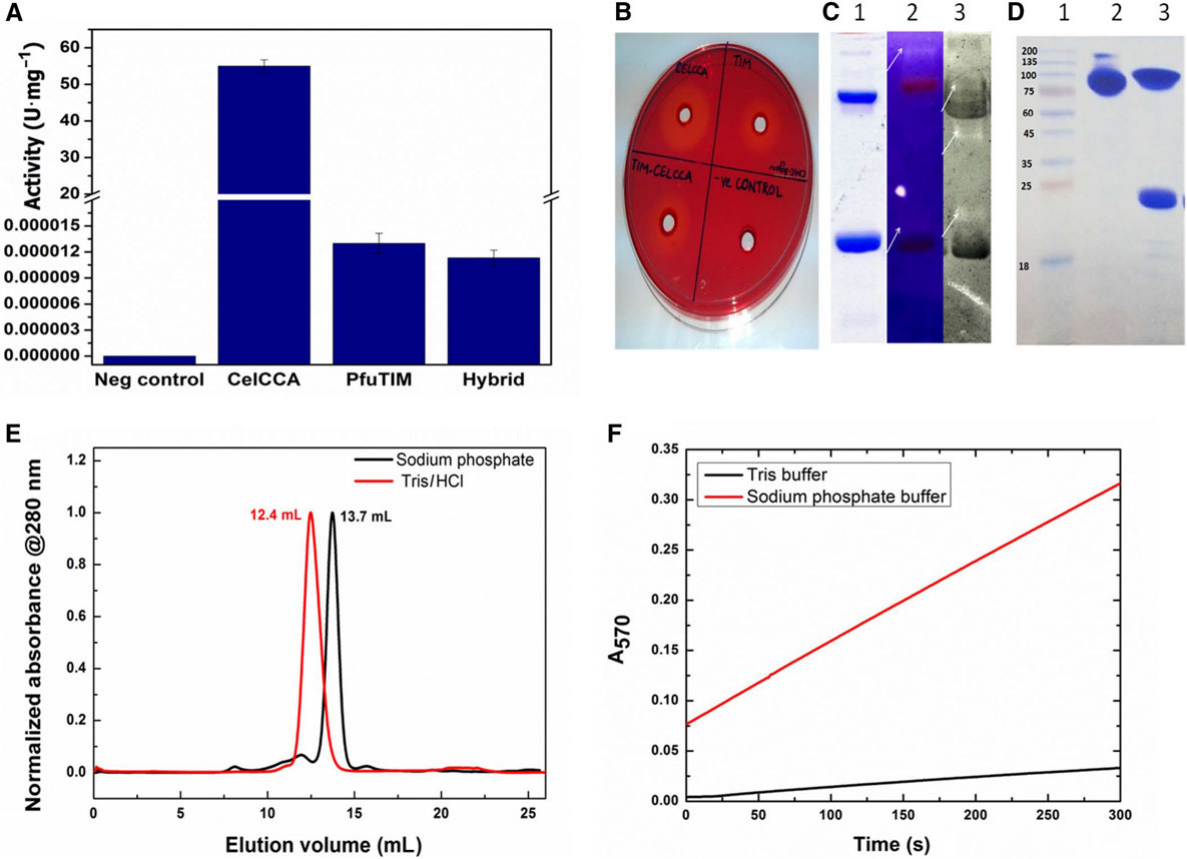
(A) Tubes representing different reactions of color development in DNSA assay; a) a true negative control (RibB from H. pylori), b) PfuTIM (a supposed negative control), c) positive control (CelCCA from *Clostridium cellulolyticum*), and d) an engineered protein (a hybrid) for which endoglucanase/cellulase activity is tested. (B) Absorbance readings taken at 570 nm for endoglucanase/cellulase activity in DNSA assay with different bars representing a true negative control (RibB from *H. pylori*), PfuTIM (a supposed negative control), positive control (CelCCA from *C*. *cellulolyticum*), and an engineered protein (a hybrid) for which endoglucanase/cellulase activity is tested. (The data represented here are taken from five independent experiments). (C) Congo Red plate assay for endoglucanase/cellulase activity with zone of clearance indicating the hydrolysis of the substrate (CM‐cellulose) in PfuTIM (a supposed negative control), positive control (CelCCA from *C*. *cellulolyticum*), and an engineered protein (a hybrid) for which endoglucanase/cellulase activity is tested. (D) Zymogram for endoglucanase/cellulase activity with zone of clearance indicating the hydrolysis of the substrate (CM‐cellulose) in PfuTIM indicated by arrows with dimer, tetramer, and octamer showing activity.

During the use of PfuTIM as a ‘presumed’ negative control in standard Miller's endoglucanase assays involving certain other known endoglucanases/cellulases, we found that the enzyme displays significant (entirely unexpected) activity as an endoglucanase. The data presented in Fig. 1A show four reactions: (a) a true negative control enzyme (a GTP cyclohydrolase called RibB, from *Helicobacter pylori*), which was not expected to show any activity/color; (b) a second ‘supposed’ negative control enzyme (i.e., the triosephosphate isomerase, PfuTIM, from *Pyrococcus furious*), which, surprisingly, showed activity/color; (c) a known positive control enzyme (a cellulase, CelCCA, from *Clostridium cellulolyticum*) expected to display activity/color on account of its being a known endoglucanase/cellulase, which showed color as anticipated, and (d) a hybrid chimeric enzyme made by fusion of PfuTIM and CelCCA. In these reactions, color was generated by the reaction of DNSA (dinitrosalicylic acid) with reducing sugars. In Fig. 1A, data from multiple experiments involving PfuTIM endoglucanase/cellulase assays are shown with error bars, presenting the activity in units/mg protein (calculated on the basis of end‐point absorbance values at 540 nm) for reactions involving the same four enzymes mentioned above. Further, detailed analysis of the observation is presented in the following section(s).

#### Plate‐based cellulase assay

Figure 1B shows results of Congo Red dye‐based plate assays in which cellulase activity was monitored visually, in terms of the development of a zone of clearance around a cup‐shaped cavity in which a solution of enzyme was deposited and incubated overnight at 37 °C. Such a zone of clearance is indicative of the digestion of cellulose in the vicinity of the cavity containing the enzyme, as it diffuses away from the cavity and digests cellulosic substrate (premixed with agar) in the vicinity of the cavity to make substrate unavailable for binding to dye. Again, PfuTIM is clearly seen to display zones of clearance indicative of degradation of cellulose.

#### Zymogram‐based cellulase assay

**Figure 2:**
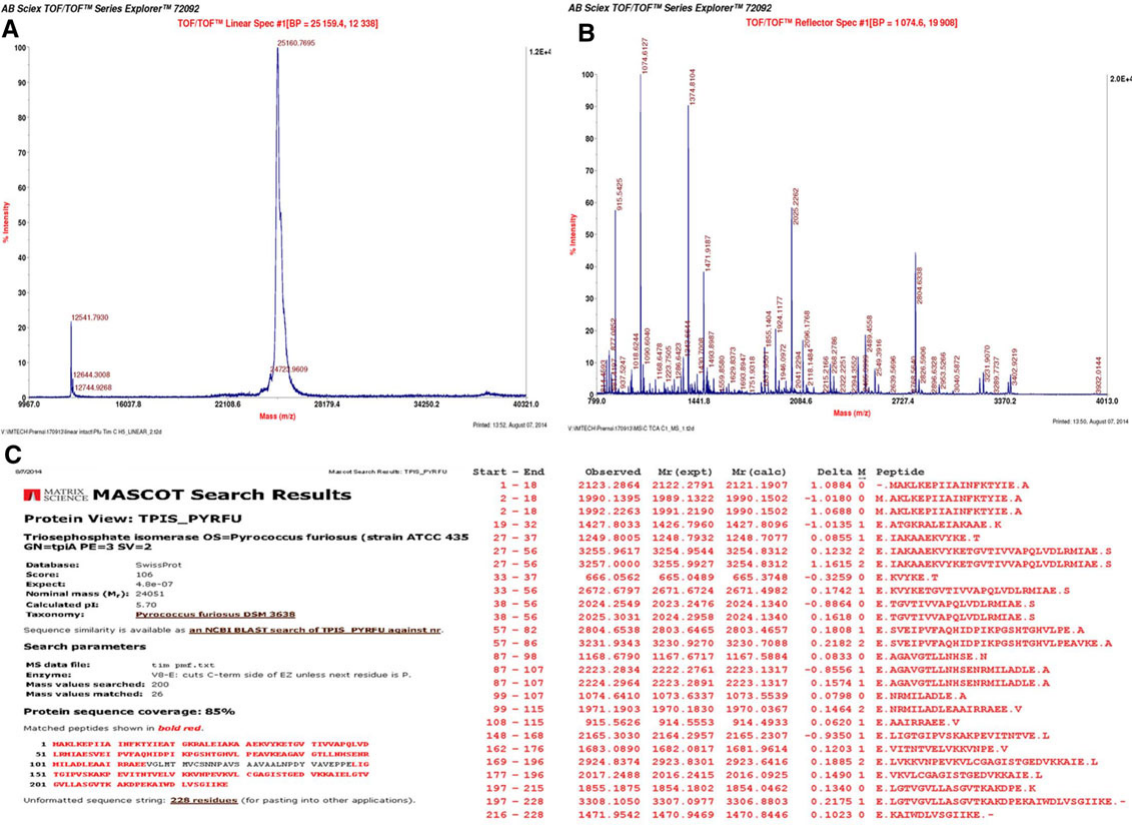
(A) Chromatogram depicting intact mass determined for PfuTIM. (B) Peptide mass fingerprint of PfuTIM (octameric species) generated after digestion of the desired band with V8 protease. (C) Prediction results for files containing the masses of the generated trypsinolytic peptides from octameric PfuTIM produced by the Mascot database server.

Finally, we also performed zymogram activity assays to additionally confirm, and directly ascribe, the observed activity to PfuTIM and not to any contaminant proteins. In these assays, PfuTIM behaves as a hyperthermophile protein, which is extremely resistant to denaturation; thus, when it is run on SDS/PAGE under standard conditions, whereas most of the protein population becomes unfolded and is seen to be electrophoresed in monomeric form, some residual population of protein is also observed to be dimeric, tetrameric or octameric, with variations in the amounts of these depending upon the kinetic stability of the population and the duration of boiling in the presence of SDS. In Fig. 1C, zymograms are shown in which clearance of substrate in the form of gel bands (i.e., clearance of associated color) can be seen. These are dependent on (a) the presence of carboxymethyl cellulose (CMC), and (b) the binding of Congo Red (which turns blue upon addition of acetate, during gel staining/destaining after electrophoresis). Where the protein in a gel band digests the CMC surrounding it, there is no Congo Red staining. In Fig. 1C, the most intense clearance is seen to be associated with the octameric form, which fails to undergo any denaturation. Some clearance is also seen to be associated with the hexameric and tetrameric forms, which have undergone partial dissociation, and occasionally with the trimeric/dimeric form. The crystal structure [PDB/RCSB ID 1HG3] of a sequence‐identical analog of PfuTIM from a related archaeon, *Pyrococcus woesei,* suggests that it is a dimer of tetramers. However, in our studies in solution involving analysis by SDS/PAGE in which the gel contains SDS but the sample loading dye does not contain SDS, akin to a native gel (Fig. 1D; showing mobility behavior), analysis by hydrodynamic volume and quaternary structural status estimation by gel filtration chromatography (Fig. 1E) and by analytical ultracentrifugation (sedimentation velocity experiments; data not shown), PfuTIM appears to be a mixture of hexamer and trimer in solution in phosphate buffer, but a mixture of tetramer and dimer in Tris buffer. In Figure 1D, lane 2 shows a trimer (~ 75 kDa) and a trace hexamer (~ 150 kDa) when there is no SDS added to the sample loading buffer, but a monomer (~ 25 kDa) and tetramer (~ 100 kDa) when SDS is added (with no boiling of the sample in either case). In Figure 1E, PfuTIM is seen to elute upon gel filtration primarily as an octamer (~ 12.4 mL) in Tris buffer and primarily as a hexamer (~ 13.7 mL) in phosphate buffer. Given these apparent differences in the quaternary structural forms of PfuTIM in Tris and phosphate buffers, it appears that the protein exists as a combination of octamer, tetramer, dimer, and monomer forms in Tris buffer. The least intense clearance in Fig. 1C appears to be associated with the monomeric form, suggesting that the endoglucanase/cellulase activity is associated with higher order quaternary structure, and not with the monomer. It may be mentioned in passing that we have established through mass spectrometry (i.e., in‐gel digestion, followed by MALDI‐TOF MS; please see Fig. 2A, B and C, showing representative intact mass, peptide mass fingerprinting, and MASCOT analysis data, respectively, for the band corresponding to the octamer population in the gel; similar data were obtained for all bands) that each of these aforementioned quaternary structural forms (i.e., bands seen in SDS/PAGE when SDS is not included in the sample loading buffer) is PfuTIM and not any other protein, or protein contaminant. Thus, in these zymograms, the activity is clearly established to be physically associated with most forms of PfuTIM itself, albeit to different degrees.

**Figure 3:**
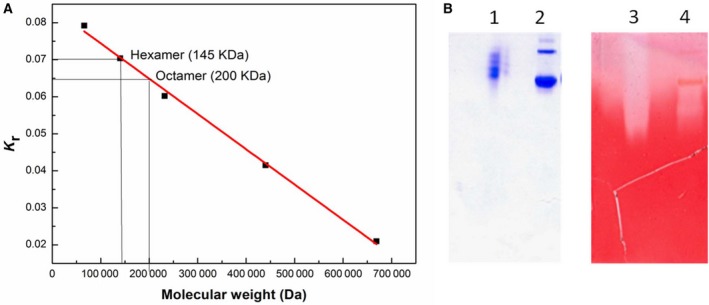
(A) Ferguson plot constructed by plotting slopes of the relative mobility of different markers on different percentages of acrylamide (5, 7.5, 10, 12.5, and 15%) in native PAGE vs the molecular weight. The slope for PfuTIM is calculated by interpolation of data for hexameric and octameric populations. (B) Zymogram for endoglucanase/cellulase activity (using 12.5% Native PAGE) with zone of clearance indicating the hydrolysis of the substrate (CM‐cellulose) in PfuTIM. Lanes 1 and 2 represent the positive control (CelCCA) and PfuTIM stained with Coomassie Blue, while lanes 3 and 4 represent CelCCA and PfuTIM stained with Congo Red.

In addition to performing zymograms with SDS/PAGE, we also carried out Ferguson plot analysis of the molecular weight (using native PAGE) for the band(s) associated with the endoglucanase/cellulase activity (Fig. 3A and B, which show the Coomassie‐stained native PAGE, and the corresponding zymogram after acetate treatment). With native PAGE too, it was possible to establish that the activity is mainly associated with two populations, an octamer and a hexamer, which create a large zone of clearance around themselves (Fig. 3A shows the Ferguson plot, while Fig. 3B shows the Coomassie Blue‐ and Congo Red‐stained native gels). Together, the data shown above establish beyond all reasonable doubt that there is an endoglucanase/cellulase activity associated with PfuTIM. This has not been noticed by us before, or reported to be the case for any TIM in the literature.

#### Synthetic substrate‐based cellulase assay

We also verified the cellulase activity of PfuTIM using a synthetic, colorimetric substrate known as cellobioside–resorufin. This substrate is degraded by cellulases to give rise to color owing to released resorufin, assessed through monitoring of increase in absorbance at 570 nm. Experiments with this substrate, performed to kinetically characterize the cellulase activity of PfuTIM, are described in a later section.

### Exclusion of the possibility of a contaminating cellulase: experiments and reasoning

As the optimal cellulase activity with a colorimetric synthetic substrate (cellobioside–resorufin) was observed at a temperature close to that of the optimal growth temperature of the mesophile organism (*Escherichia coli*) in which PfuTIM was produced in recombinant form (i.e., 37 °C), we examined further the remote possibility that the cellulase activity derives from some unknown contaminant protein inadvertently cosourced with PfuTIM from *E. coli* during purification. We excluded this particular possibility in the following (three) ways, based partly on re‐examination of the data already presented above, and partly on new experiments involving heating of the protein.

#### Two‐stage purification

PfuTIM purified through affinity methods (using IMAC chromatography) was repurified by gel filtration chromatography, to separate proteins on the basis of their hydrodynamic radii. As described in the above section, in Tris buffer, PfuTIM behaves (and elutes from gel filtration chromatography columns) primarily as an octamer. A purification table is included as Table 1. The rationale behind this approach was that any contaminating protein not physically associated with PfuTIM would separate away from PfuTIM during gel filtration, while a contaminating protein, which seems to be physically associated with PfuTIM, would be expected to alter the elution volume of PfuTIM significantly enough to be noticeable during gel filtration chromatography. We found activity to be present only in the protein fractions eluting at the elution volume at which PfuTIM elutes, indicating that there is neither any free contaminating protein, nor any PfuTIM‐bound contaminant protein responsible for the observed endoglucanase/cellulase activity. The alternative explanation—namely that a contaminant coelutes with PfuTIM in both chromatographic steps—is too unlikely to be given any further consideration.

**Table 1:** Purification table for PfuTIM's endoglucanase activity

Fraction	Volume (mL)	Total protein (mg)	Specific activity (nmoles·min^−1^·mg^−1^)	Fold purification	Percentage yield
Cell lysate	32	256	57.71	1.0	100
Flowthrough	30	180	30.72	0.532	70.31
Wash	30	48	59.2	1.02	18.75
IMAC elution	2	12	3.96	0.067	4.68
After gel filtration	3	7.2	64.71	1.12	2.81

#### Thermal deactivation of potential contaminants

**Figure 4:**
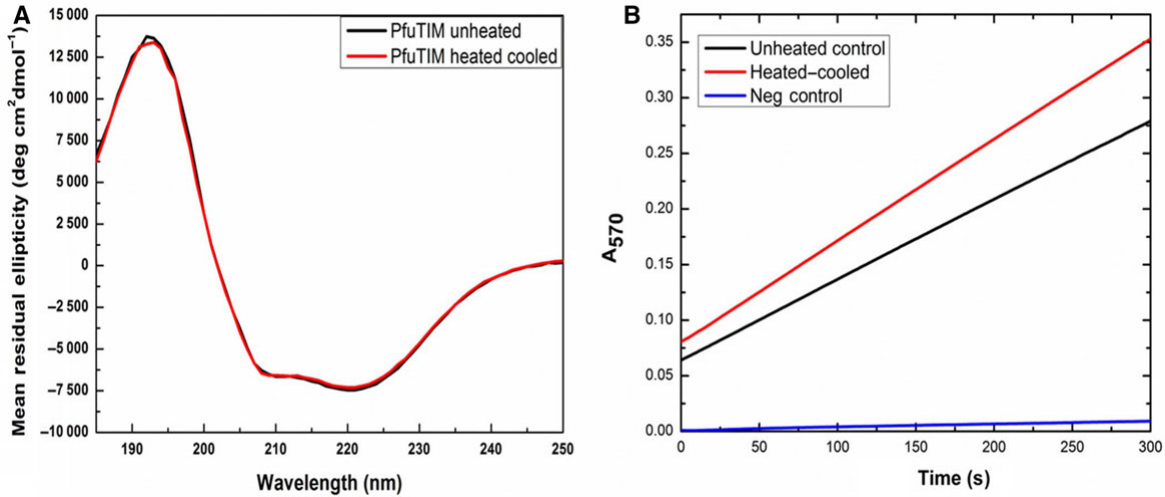
(A) Far‐UV CD spectra of PfuTIM before and after heating and cooling to 90 °C. The raw ellipticity is shown without conversion to mean residue ellipticity (MRE) to emphasize the overlap of spectra. (B) PfuTIM shows an increased activity at 37 °C when the heated–cooled protein was used (red) as compared to the unheated control (black).

The purified PfuTIM protein was heated at 90 °C for 1 h and cooled to room temperature before activity assays were performed. During the entire experiment, the far‐UV CD spectrum of the protein was recorded at 222 nm to confirm that PfuTIM underwent no denaturation through heating and cooling. Figure 4B, presenting mean residue ellipticity (MRE) measurements as a function of spectral wavelength, demonstrates that there is no alteration in PfuTIM's secondary structure upon heating to 90 °C, as would indeed be expected for any ultra‐thermostable hyperthermophile‐derived protein. On the other hand, any contaminating protein derived from *E. coli* would definitely be expected to become completely and irreversibly denatured upon such treatment, as no *E. coli‐*derived protein is known (or expected) to survive exposure to such temperatures and then undergo any substantive, and facile, refolding to native form. Figure 4B shows that, in fact, the heated–cooled protein shows increased endoglucanase/cellulase activity after such treatment, rather than a decreased level of activity (or no activity) at 37 °C. The *y*‐axis in the panel indicates changes in absorbance at 570 nm, and the rising absorbance (positive slope) is indicative of activity giving rise to product (i.e., released resorufin) with time. An increase in activity can only be explained if it is attributable to a hyperthermophile protein that undergoes no denaturation (due to high thermodynamic and kinetic stability). Notably, such a recombinant protein produced in *E. coli* growing at 37 °C would be expected to adopt native structure even more efficiently (molecule by molecule) at 90 °C on account of its having been designed to fold at such high temperatures by evolution, in a hyperthermophile genome/proteome. It could thus display a higher amount of activity after exposure to 90 °C, instead of a lower amount of activity (owing to denaturation, which would be compatible with the behavior expected of any mesophile‐derived protein from *E. coli*). Thus, any *E. coli ‐*derived contaminant is established to be unlikely to be responsible for the observed activity. However, it is not clear yet why PfuTIM should have displayed any endoglucanase/cellulase activity at mesophilic temperatures.

#### No such activity in a different mesophile‐derived TIM, derived from *Saccharomyces cerevisiae*

A different protein of identical nature and activity but of nonhyperthermophile origin, that is, a recombinant yeast TIM bearing the sequence of TIM from *Sachharomyces cerevisiae*, was similarly expressed and purified, using the same conditions earlier used for the expression and purification of PfuTIM. This ‘control’ yeast TIM was examined for the presence of cellulase activity. However, none could be observed (data not shown), suggesting both (a) that *E. coli* contaminants ordinarily copurifying with a 6xHis‐tagged protein are not responsible and (b) that a different TIM from *S. cerevisiae* does not display the observed endoglucanase/cellulase activity.

#### No such activity in a different hyperthermophile‐derived TIM, TonTIM, derived from *Thermococcus onnurineus*

We have cloned and produced a PfuTIM‐related TIM from *Thermococcus onnurineus,* TonTIM, sharing approximately 92% identity with PfuTIM 4. Both PfuTIM and TonTIM have been shown by us previously to be superperfect enzymes, with activities at high temperatures that are at least an order of magnitude higher than anything reported for a mesophile‐derived TIM 5. We examined TonTIM, purified in an identical manner to PfuTIM, to find out whether it displays any endoglucanase/cellulase activity. TonTIM showed no detectable endoglucanase/cellulase activity whatsoever (data not shown).

#### Association of activity with PfuTIM bands in zymograms

The observation of activity in a zymogram, as already described above for Fig. 1D, definitely indicates that a contaminant cannot be responsible, as each of the bands in the PfuTIM sample (corresponding to the octameric, tetrameric, and monomeric forms of TIM) is seen in the zymogram. These bands have been independently determined in our laboratory to be PfuTIM, that is, PfuTIM associated with different quaternary structural states, failing to be denatured in SDS/PAGE on account of lack of SDS in the sample loading buffer and lack of boiling of sample. The identities of the different quaternary structural forms were established through in‐gel trypsinolytic digestion and MALDI‐TOF MS analysis, as already mentioned, and shown (Fig. 2A, B and C, presenting representative data for only the octamer form).

Therefore, it stands fully established that the endoglucanase/cellulase activity owes to PfuTIM and not to any contaminant. Further, it is established that the activity is shown mainly by the native folded, homomultimeric forms of PfuTIM (i.e., the octameric form and also the tetrameric and dimeric forms). Activity is enhanced through heating to a temperature (90 °C), which causes no denaturation. Lack of heating in the presence of SDS causes no decrease in activity; however, when samples are boiled in the presence of SDS and all proteins are converted to the monomeric SDS‐bound form, no activity is seen to be associated with the monomer alone in the zymogram (data not shown), presumably because of subunit dissociation associated with chain unfolding. Collectively, these results support the likely existence of a novel endoglucanase/cellulase activity associated with PfuTIM.

### pH and temperature profiling of PfuTIM's endoglucanase/cellulase activity

**Figure 5:**
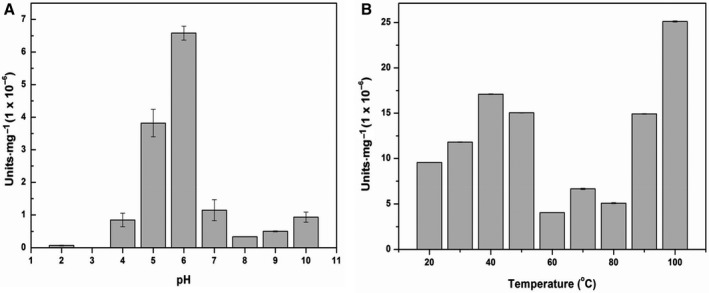
(A) Absorbance vs pH profile of PfuTIM collected at 35 °C. An average of three independent readings is plotted. (B) Absorbance vs temperature profile of PfuTIM at pH 6.0. An average of three independent readings is plotted.

Figure 5A and C, respectively, show data for multiple experiments measuring changes in PfuTIM's endoglucanase/cellulase activity as a function of pH and temperature, in units/mg protein, measured with the synthetic colorimetric substrate, cellobioside–resorufin. The cellulase activity is clearly seen to peak at a pH of 6.0 (Fig. 5A). The determined temperature range of optimal cellulase activity in PfuTIM was found to distribute into two peak temperature ranges: one between 30 and 40 °C and the other approaching 90–100 °C (Fig. 3B). As PfuTIM is an enzyme derived from the genome of a hyperthermophile, *P. furiosus*, which shows optimal growth at ~ 102 °C, it is expected to show activity at a temperature close to ~ 100 °C. Therefore, the activity observed at or close to ~ 100 °C is understandable. It is intriguing that there is an additional activity peak in the range of 30–40 °C. It is difficult to explain this activity in the lower temperature range, which could potentially relate to some basal survival mechanism for the organism at colder water temperatures. At this point of time, we are unable to speculate upon what such a mechanism could be. However, at the same time, this observed activity at ‘mesophile’ temperatures is potentially useful, because it allows us to kinetically characterize the enzyme using a synthetic substrate, cellobioside–resorufin, which behaves well in the mesophile temperature range but undergoes auto(hydro)lytic degradation at temperatures above 80 °C. As an aside, we wish to mention here that in various previous studies of the structural stability of PfuTIM previously performed and reported by us 3, 5, 6, 7, 8, we had never actually examined TIM activity in PfuTIM because of the requirement of a coupled assay dependent on the availability of another hyperthermophile protein. Recently, however, through a different assay relying on measurement (using circular dichroism) of the depletion of the d‐form of G3P in a racemic mixture of d‐form and l‐form of G3P, we have established that PfuTIM is a superperfect enzyme with inordinately high TIM activity 5.

### Kinetic characterization of the endoglucanase/cellulase enzymatic activity

#### Activity assays using cellobioside–resorufin as substrate

**Figure 6:**
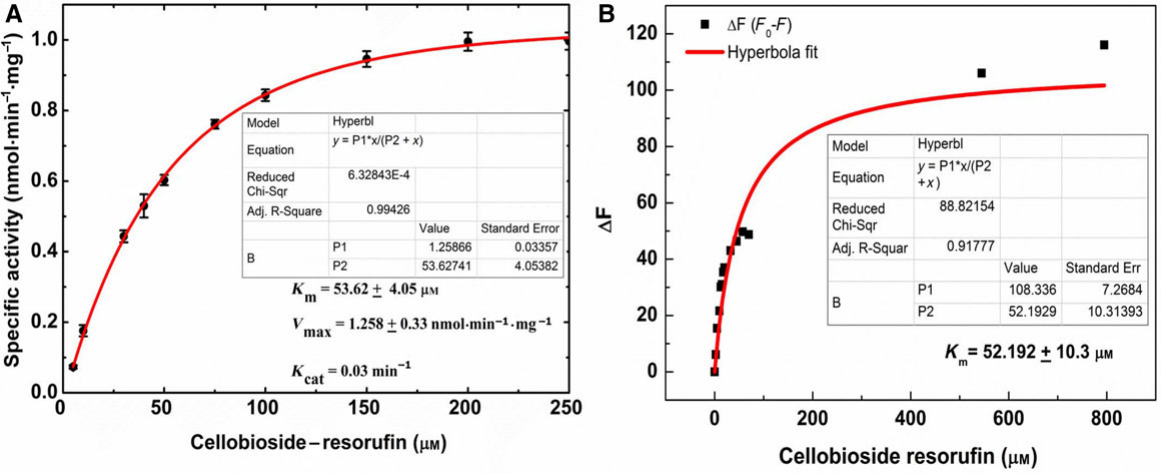
Michaelis–Menten plot for PfuTIM's endoglucanase activity. The plot represents specific activity vs cellobioside–resorufin concentration. (B) Differential fluorescence indicative of quenching of intrinsic tryptophan fluorescence of PfuTIM (due to binding of cellobioside–resorufin) as a function of increasing concentration of cellobioside–resorufin (substrate). The concentrations of cellobioside–resorufin used are 0, 0.025, 0.05, 0.1, 0.125, 0.15, 0.175, 0.2, 0.325, 0.45, 0.575, 0.7, 1.45, 2.95, 5.45, and 7.95 8 μm.

We characterized the endoglucanase/cellulase activity of PfuTIM using a fluorescence assay‐based kit (see materials and methods) utilizing cellobioside–resorufin as a substrate. In this assay, the release of the resorufin, by the activity of the enzyme on the substrate, gives rise to color, which can be monitored at 570 nm to monitor the progress of the reaction. The Michaelis–Menten plot of the reaction is shown in Fig. 6A, based on data from eight independent experiments. It is evident that the PfuTIM enzyme displays standard Michaelis–Menten kinetics even as an endoglucanase/cellulase, with the velocity of the reaction (shown as specific activity on the *y*‐axis) initially increasing with increasing substrate concentration and displaying a ‘plateau’ above a certain concentration (~ 200–250 μm) of the substrate. The kinetic parameters were determined as follows: The K_m_ was determined to be ~ 53.62 ± 4.05 μm in an absorbance‐based assay using cellobioside–resorufin as substrate (Fig. 4A), and ~ 52.19 ± 10.3 μm in a fluorescence quenching‐based assay using the same substrate (Fig. 6B), with reasonably tightly distributed observations over eight experiments in Fig. 4A, and one representative curve shown in Fig. 6B. The V_max_ was determined to be 1.258 ± 0.033 nmol^−1^· min^−1^·mg. The k‐cat was determined to be 0.03 min^−1^.

#### Competitive inhibition of cellulose activity with known TIM inhibitors

**Figure 7:**
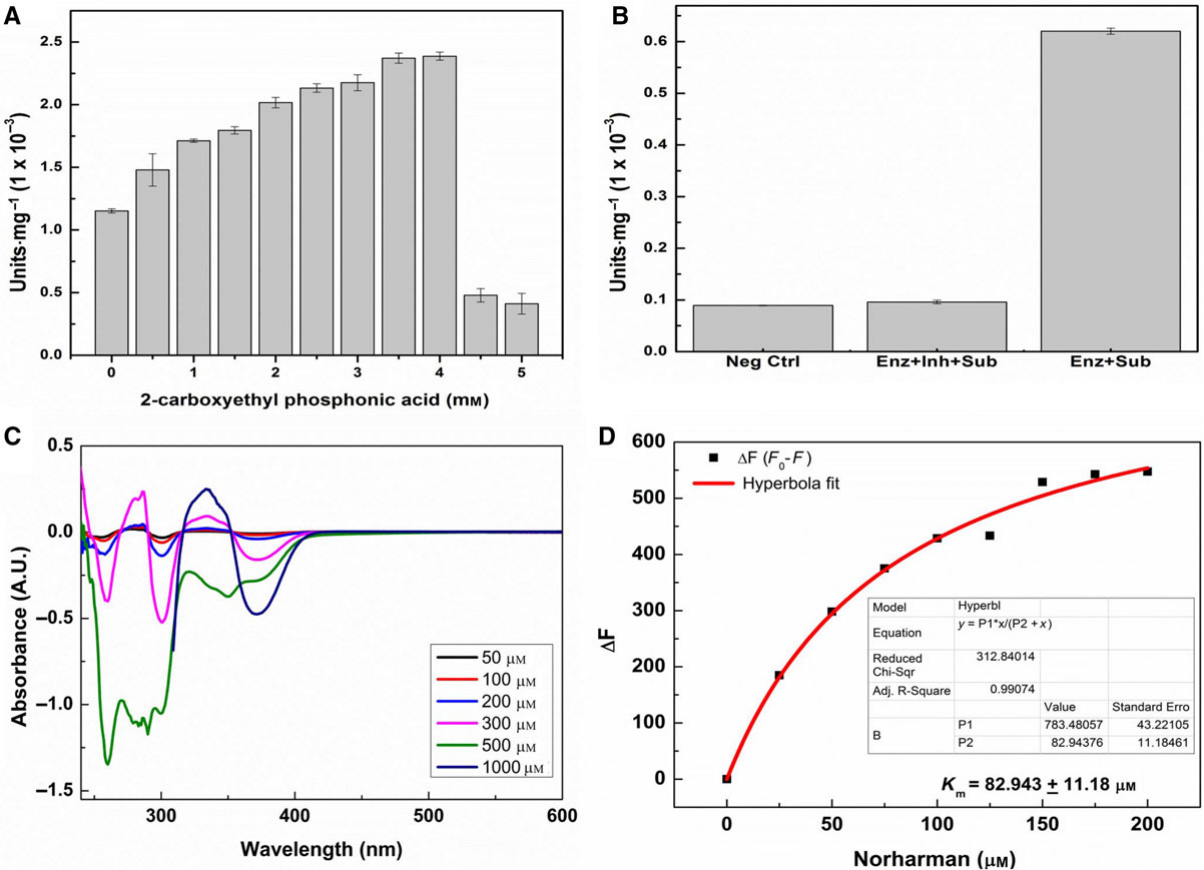
(A) Competitive inhibition of PfuTIM's endoglucanase/cellulase activity with 2‐carboxyethyl phosphonic acid/3‐phosphopropanoic acid (3‐PPA). The concentrations of 3PPA used were 0, 0.5, 1.0, 1.5, 2.0, 2.5, 3.0, 3.5, 4.0, 4.5, and 5 mm. (B) Competitive inhibition of PfuTIM's endoglucanase/cellulase activity with beta‐carboline/norharman. The concentration of norharman used was 25 μm. (C) Difference absorption spectra of PfuTIM's interaction with different concentrations of norharman. The concentrations of norharman selected were 50, 100, 200, 300, 500, and 1000 μm. (D) Differential fluorescence indicative of quenching of intrinsic tryptophan fluorescence of PfuTIM (due to binding of inhibitor) as a function of increasing concentration of norharman (inhibitor). The concentrations of norharman used were 0, 25, 50, 75, 100, 125, 150, 175, and 200 μm.

There are two kinds of inhibitors of TIM: (a) those which are structural analogs of TIM's natural substrate, G3P, such as 3‐phosphopropanoic acid (3‐PPA), also known as 2‐carboxyethyl phosphonic acid, which bind to the same site as G3P 9 and (b) those which are not structural analogs of G3P, but which still display TIM activity inhibition by binding to a different (not yet identified) site present on all TIM enzymes, such as beta‐carbolines, or norharman 10. We used both 3‐PPA and norharman with PfuTIM to examine whether known TIM activity inhibitors also inhibit the observed endoglucanase/cellulase activity. This was done to address the question of where cellulose, or cellobioside, binds initially. Notably, sharing of sites with substrate analog inhibitors would result in some steric inhibition of one, or the other, activity, while with nonsubstrate analog inhibitors this would not be expected to occur, especially if such inhibitors were to somehow act allosterically. Figure 7A shows that 3‐PPA, which is an analog of G3P, significantly enhances endoglucanase/cellulase activity in PfuTIM, instead of showing any ability to inhibit such activity, as a function of increasing concentration, progressively up to a concentration of 4 mm (however, with a sharp and difficult‐to‐explain decrease in activity in the presence of higher concentrations). This behavior potentially owes to action at a distance and occurs because 3‐PPA binding occurs at a site different from the one where cellobioside–resorufin binds. Figure 7B shows that the other (nonsubstrate analog) inhibitor, norharman, however, definitively inhibits endoglucanase/cellulase activity in PfuTIM.

Combined, these results indicate that the endoglucanase/cellulase and TIM activities very likely do not owe to the same site, as 3‐PPA (the substrate analog of G3P) does not give rise to any discernible steric inhibition of cellulose or cellobioside binding and hydrolysis; rather, it appears to promote the endoglucanase/cellulase activity. Interestingly, the norharman‐binding site, which has not been structurally determined thus far for any triosephosphate isomerase, is thought to engage in allosteric interactions with the G3P binding site and probably does not have any shared residues with the cellulose‐binding site. With norharman, complete inhibition of endoglucanase/cellulase activity is seen, suggesting an unexpected and fortuitous coincidence, or partial overlap, of the norharman‐binding site with the site responsible for endoglucanase/cellulase activity, as norharman binding is able to inhibit binding and hydrolysis of cellobioside–resorufin. To further confirm norharman's physical binding to PfuTIM, we carried out difference absorption spectroscopy experiments involving both molecules. Figure 7C clearly shows marked difference absorption spectra varying with norharman concentrations, establishing that norharman binds to PfuTIM.

### Fluorescence quenching studies with norharman (inhibitor) and cellobioside–resorufin (substrate)

PfuTIM contains a single tryptophan residue (intrinsic fluorophore) present at the C‐terminal end of the (β/α)_8_ barrel structure. This residue is quite exposed and faces the solvent. Quenching of intrinsic tryptophan fluorescence was monitored (in the range from 300 to 400 nm, with excitation at 295 nm) as a function of increasing inhibitor/substrate concentrations to examine any potential effects of norharman binding upon PfuTIM's intrinsic fluorescence. A decrease in fluorescence intensity (or quenching) of tryptophan fluorescence was observed with the increasing concentration of norharman. The resulting difference in intensity was determined and plotted as a function of norharman concentration, with hyperbolic fitting and determination of KD (~ 83 μm), as shown in Fig. 7D. Comparing the KD values obtained for quenching by the cellulose chain or cellobioside (Fig. 4B) and by the inhibitor, norharman (Fig. 5D), it appears that binding of the cellulose chain or cellobioside is either slower, or occurs with lesser affinity, than with norharman.

### Effect of different buffer conditions on activity

PfuTIM purified in Tris buffer of pH 8.0 does not show any endoglucanase/cellulase activity. When the protein is purified in sodium phosphate buffer of pH 8.0, however, endoglucanase/cellulase activity is seen. Interestingly, if purification is first done in phosphate buffer and then the protein is transferred into Tris buffer, the Tris ions are not able to inhibit activity, and the protein shows comparable activity to that of protein purified in phosphate buffer and retained in phosphate buffer. Although we have shown in Fig. 1E that there are differences in the hydrodynamic volumes and, therefore, in the likely quaternary structures of PfuTIM in Tris and phosphate buffers, which could be responsible for these effects, we do not have any immediate, or facile, explanations for these interesting observations. Thus, we were able to observe activity in Tris buffer, for example, in the zymograms, but all kinetic characterization was done in phosphate buffer.

### Further examination of the effects of heating and cooling on the endoglucanase/cellulase activity

**Figure 8:**
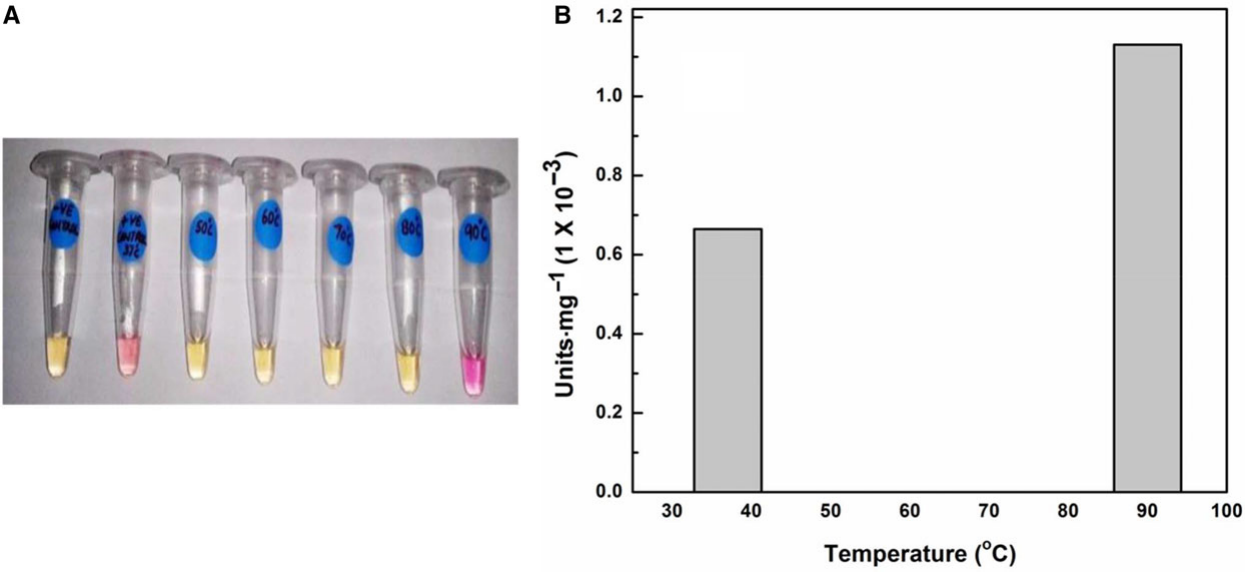
(A) Tubes representing reactions to see color change (pink) due to hydrolysis of substrate (cellobioside–resorufin) for checking endoglucanase/cellulase activity with PfuTIM heated up to 50, 60, 70, 80, and 90 °C. The activity assay was set up at 37 °C. (B) Absorbance readings taken at 570 nm to check endoglucanase/cellulase activity with PfuTIM heated up to 50, 60, 70, 80, and 90 °C. The activity assay was set up at 37 °C.

As already reported, heating to 90 °C and cooling resulted in enhanced activity. However, when heating was done up to temperatures lower than 90 °C, followed by cooling, activity appeared to be lost. When the protein was incubated for an hour up to the temperatures of 50, 60, 70, 80, and 90 °C and then cooled to 37 °C and the activity assay was set up at 37 °C, no activity was observed in the reactions in which the protein was incubated at 50, 60, 70, and 80 °C, but in the last of these reactions (i.e., where PfuTIM was incubated at 90 °C and then cooled) enhanced activity was seen. The data are presented in Fig. 8A and B. The data are shown visually in Fig. 8A. Our interpretation of these data is that there are effects on the enzyme's microstructure upon heating to 50, 60, 70, and 80 °C, which are not reversed upon cooling. However, at the higher temperature of 90 °C, the endoglucanase/cellulase active site of the enzyme, which is probably present at subunit interface(s), settles once again into a structure very much like that in the enzyme purified from *E. coli* without any heating. Given that PfuTIM must have evolved to act optimally as TIM at a temperature close to 90–100 °C, it would not be surprising if the enzyme's folding were to be further refined through exposure to temperatures in this range to yield a more active structure, even for assays measuring activity at lower temperatures, upon cooling of enzyme exposed to 100 °C. We realize that the above does not constitute a very good clarification of the relationship between folding and temperature. This is because we are only trying to emphasize that an enzyme, which has evolved to fold and function correctly at a temperature close to (or above) 90 °C, could possibly require exposure to such a regime of temperature before any required minor (subtle) adjustments of side‐chain packing would occur to allow activity to rise to detectable levels. Unfortunately, this is a conceptual issue, which cannot be elaborated upon further in any detailed structural terms, at this point in time. It may be noted that re‐examination of heated and cooled protein showed no changes whatsoever in the chromatographic (gel filtration) profiles of samples, relative to unheated samples; of course, this was expected, given the extraordinary hyperthermal stability of PfuTIM 3, 5, 6, 7, 8.

### Effect of mutations on the activity: Indications of a ‘second site’ for cellulase activity

**Figure 9:**
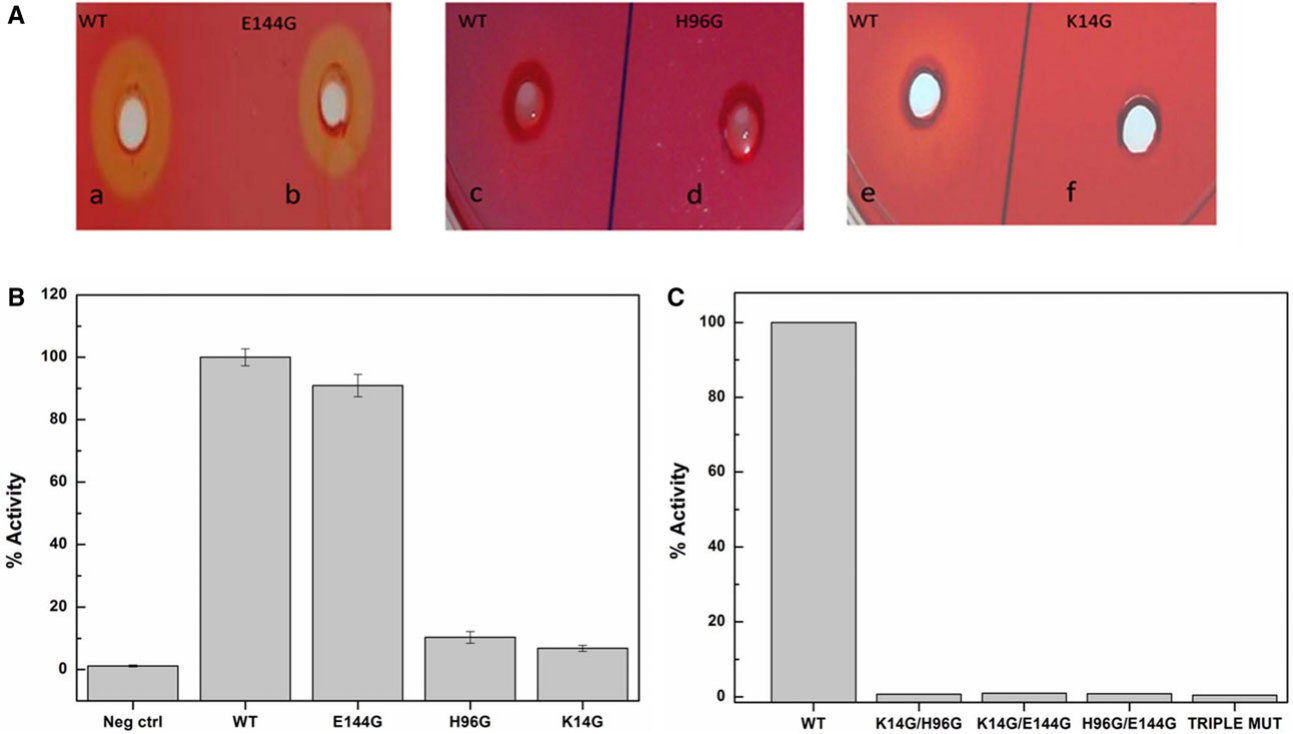
(A) Congo Red plate assay showing a prominent zone of clearance after overnight incubation at 37 °C in wild‐type (a, c, and e) and E144G, (b) whereas no zone of clearance was visible in H96G (d) and K14G (f). (B) Absorbance readings taken at 570 nm with the reactions being carried out at 37 °C. The wild‐type PfuTIM and E144G mutant was incubated for an overnight reaction, whereas H96G and K14G mutants were incubated for a longer duration (15 days). Each datum is an average of three independent experiments. (C) Absorbance readings taken (for double and triple mutants) at 570 nm with the reactions being carried out at 37 °C.

Our studies involving the use of the two inhibitors, 3‐PPA and norharman, suggest that the residues involved in catalysis of cellulose hydrolysis are different from those required for TIM activity. We decided to verify this further by examining the effect of mutating key catalytic residues of TIM activity, namely the triad of residues, Lys14, His96, and Glu144, upon the observed endoglucanase/cellulase activity. All three residues were individually replaced by glycine. As is evident from plate assays shown in Fig. 9A, E144G is almost as active as wild‐type protein, while K14G and H96G do not show activity over the same durations of incubation. Whereas the zones of clearance seen (with different sizes and intensities) do provide some crude quantitative idea about differences in activity between the mutants and the wild‐type protein, further quantitative differences in activity can also be seen in Fig. 9B and C, where percentages of activity with respect to the wild‐type protein are plotted, for assays with cellobioside–resorufin. It should be noted that when the K14G and H96G mutants were incubated with substrate for 15 days at 37 °C and the absorption of the reaction was monitored spectrophotometrically at 570 nm, activity was seen in the other two mutants as well, as has been shown in Fig. 9B (i.e., the days of incubation are different for the K14G/H96G mutants and the E144G mutant). The observed activity suggests that there is no crippling of the basic chemistry involved in the cellulose hydrolysis, upon mutation of any of the three residues critically required for TIM activity. These results, and especially the one involving the E144G mutation, indicate that the G3P binding site is not the same as the site responsible for the endoglucanase/cellulase activity, as the glutamate residue, E144G, plays a critical role in TIM's activity as a triosephosphate isomerase. The lower activity in the K14G and H96G mutants probably owes to a subtle effect on the structure of the enzyme, felt at the endoglucanase/cellulase site. Double mutants showed negligible activity, as shown in Fig. 9C. With the triple mutant in which all three residues responsible for TIM activity were mutated, again only negligible (i.e., barely detectable) activity could be seen. The likely reason is an ‘action‐at‐a‐distance’ effect of some changes in structure at the TIM site, which are felt at the cellulase site, impacting upon its activity, as the site responsible for TIM's activity and the site of norharman binding (which appears to overlap with the site for cellulose binding) appear to respond to each other.

### Isothermal titration calorimetric studies of cellulose binding to PfuTIM

**Figure 10:**
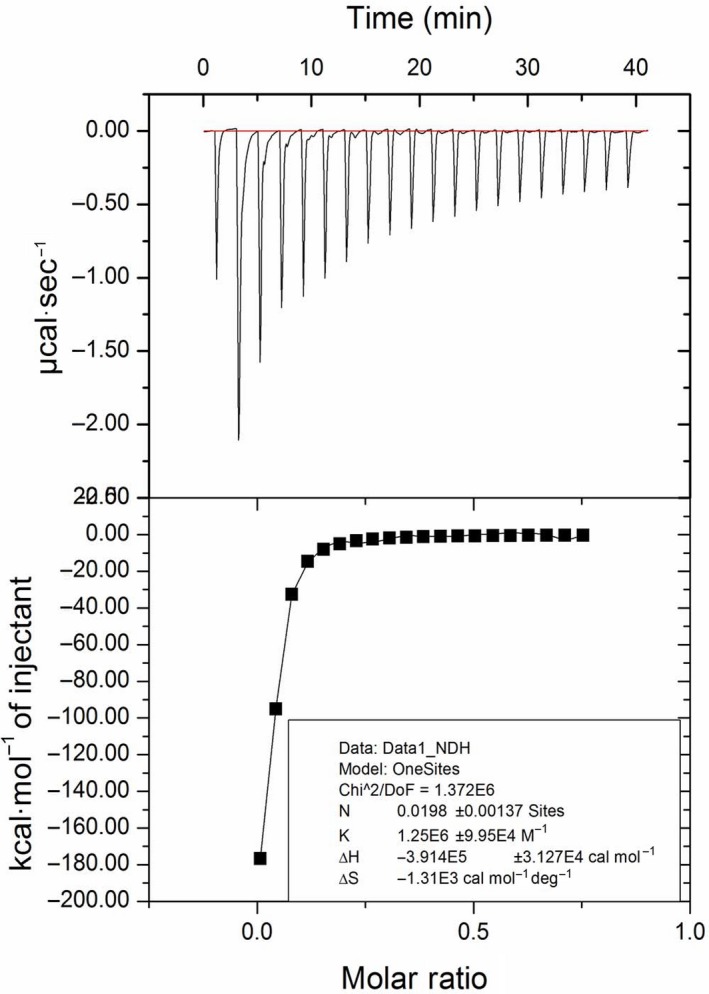
Isothermal titration calorimetry thermograms for the interaction of PfuTIM with carboxymethyl (CM) cellulose. The top panel in the figure shows the raw data for successive injections, and the bottom panel shows the fitting and analysis of interactions.

To further establish the existence of endoglucanase activity and establish the binding of CM‐cellulose to PfuTIM, we employed isothermal titration calorimetric (ITC) measurements in which heat changes were monitored, while a solution of CM‐cellulose (144 μm, stock concentration) was added to the reservoir/cell containing PfuTIM (40 μm). The thermogram obtained was subjected to buffer subtraction and fitted in a binding model showing one molecule of the enzyme to be binding to ~ 0.02 molecules of CM‐cellulose, which is a polymer of glucose of an average molecular weight of 90 000 Da, corresponding to an average chain length of 500 glucose units as seen in Fig. 10. An inset in the figure presents details of the determined binding parameters.

## Discussion

We have demonstrated beyond reasonable doubt that PfuTIM, the TIM enzyme of *P. furiosus*, displays endoglucanase activity. The activity peaks not only at the expected high temperature (for an enzyme encoded by the genome of a hyperthermophile archaeon), but also at the lower temperature of ~ 40 °C. We have also shown that the new activity does not owe to the active site responsible for the interconversion of G3P and DHAP. We showed this using the twin strategies of examining the actions of known TIM inhibitors, and effects of mutations on glycine‐substituted active site residue mutants. Although, out of the three active‐site residue mutants, two resulted in a dramatic reduction in endoglucanase/cellulase activity, significant activity could still be observed at measurable levels using other methods/assays and longer durations of incubation. The fact that the mutation of the third critical residue, a glutamate, had no measurable effect on activity indicates that even if some residues are shared between the TIM and endoglucanase/cellulase activity‐bearing sites, the sites are not absolutely identical. The probability of the sites being different is also higher because of the result that norharman (which does not bind to the standard TIM active site) completely abolishes the endoglucanase/cellulase activity, whereas 3‐PPA (an inhibitor which binds to the standard TIM active site) shows no measurable inhibition of endoglucanase/cellulase activity. Rather, over a significant range of concentrations (0.5–4.0 mm), an increase in endoglucanase/cellulase activity is observed in the presence of this inhibitor of TIM activity.

If this enzyme does indeed have two activities, two questions immediately arise. (1) Are any other TIMs, or TIM barrel proteins known, which have two activities? (2) Why does the second activity exist? For the first question, the answer is in the affirmative. Where TIM barrel fold proteins are concerned, which are not themselves triosephosphate isomerases, instances are known of enzymes displaying more than one activity 11, 12; however, no triosephosphate isomerases have thus far been shown to possess a second activity. As regards the second question, it may be noted that the metabolic processes of the anaerobic hyperthermophilic archaeon, *P. furiosus,* are considered to have evolved somewhat differently from the metabolic processes of most modern bacteria.

Several metabolic processes occur differently in *P. furiosus* 13. Two important metabolic differences relate to the availability and utilization of glucose, especially through utilization of cellulose as a carbon source. Firstly, *P. furiosus* is reported to have a modified form of the Embden–Meyerhof (EMP) pathway, which is critical for survival of single‐celled organisms lacking mitochondria, because glycolysis is the primary source of ATP 13, 14. Secondly, although *P. furiosus* is capable of utilizing chitin, laminarins, and other complex carbohydrates as a source of glucose like most prokaryotes, there are some differences. To utilize complex extracellular carbohydrates, an organism must do three different things: (a) secrete one or more enzymes capable of degrading the carbohydrate into smaller fragments, (b) possess membrane protein machinery for import of such fragments into the cytoplasm, and (c) possess intracellular enzymes capable of degrading the fragments into glucose. In *P. furiosus*, two secreted enzymes, EglA and LamA, degrade β‐linked glycans and extracellular cellulose. EglA is specific for cellulose 4, 15, 16, and LamA acts preferentially on laminarins while showing only marginal activity upon cellulose 16. An ATP‐utilizing ABC transporter also exists in *P. furiosus* for import of cellobiose (as well as cellotriose, cellotetraose, and cellopentaose); however, to degrade cellobiose or cellotriose, *P. furiosus* appears to possess only a single intracellular beta‐glucosidase, Bgl, which is reported to display preferential activity on laminarin‐derived (but not cellulose‐derived) fragments 4, 17. The bgl and lamA genes occur within the same gene cluster and the encoded enzymes are expressed in concert (to degrade β‐1,3 linkages in β‐1,3 laminarin and mixed β‐1,3‐1,4 linked glucans) upon induction by β‐1,3 glucans 4, 11.

Bgl displays 2.5 times higher activity with β‐1,3 laminaribiose, in comparison with β‐1,4 cellobiose 4. This raises a question. How are imported cellulose‐derived fragments degraded rapidly enough within *P. furiosus* to generate sufficient quantities of glucose to feed the organism's glycolytic pathway if, and when, the organism grows on cellulosic substrates? One theoretically viable solution would be for *P. furiosus* to have an additional (as yet unidentified) enzyme performing the task of degrading cellobiose and higher order fragments of cellulose, within its cytoplasm. The genome of *P. furiosus*, however, appears not to encode any other identifiable intracellular enzyme predicted (by bioinformatics analyses) to degrade either cellobiose, or longer cellulosic sugars, although it remains conceivable for such an enzyme to be encoded by a gene of as yet unknown (i.e., unassigned) function from the organism's genome. As there is currently no known candidate enzyme, which can degrade cellulose‐derived fragments imported into the cytoplasm of *P. furiosus*, the possibility must be considered that the said function is performed by another enzyme, which ordinarily performs a different function. Such an enzyme could potentially be a glycolytic pathway enzyme, which seems to be imbued with an additional (endoglucanase/cellulase) activity; in fact, this would be especially interesting if it were to somehow correlate with the fact that the organism's EMP pathway is modified, and different from those of other organisms. Is PfuTIM then the glycolytic pathway enzyme that is imbued with the additional endoglucanase/cellulase activity? Our results certainly suggest that this is possible.

It would be interesting to describe the discovery of such an enzyme as an outcome of the above discussion and analysis. However, as is often the case in science, the chronology of events involved a discovery being made by accident, followed by its post facto scrutinization and rationalization (as already mentioned in the introductory section). As *P. furiosus* has known arrangements for importing cellobiose into its cytoplasm, using ABC‐type transporters 18, we would like to speculate that it is possible that PfuTIM doubles as a ‘generator’ of glucose from cellobiose when glycolysis slows down for the need of glucose, feeding glucose into the initial reactions of glycolysis when it is not engaged in using G3P as a substrate. As there would be no need for the enzyme to work as an endoglucanase/cellulase when enough glucose is present in the cytoplasm, one might anticipate that G3P and cellobiose would both regulate the enzyme's activities inversely, with respect to the use of each as substrate, and this would necessitate the existence of an allosteric interaction between two different binding sites, one for cellobiose (or indeed any cellulose) and the other for G3P. Our results support this possibility.

**Figure 11:**
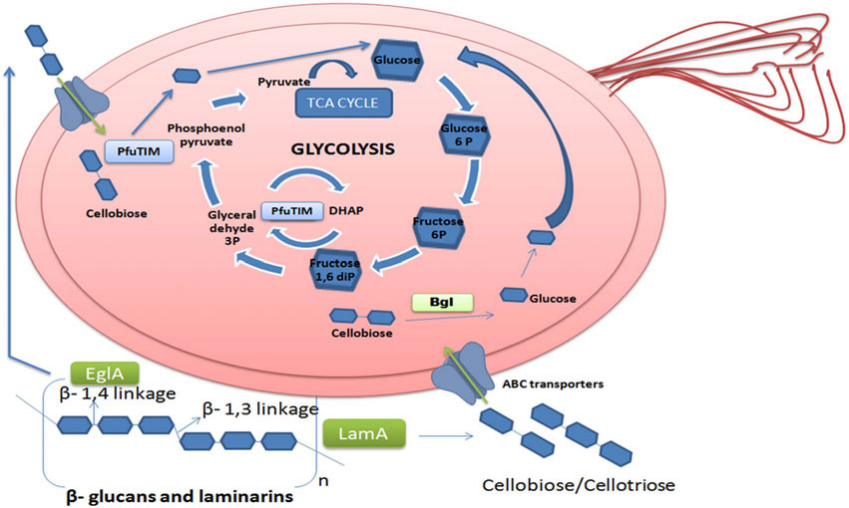
A schematic summarizing the conclusions drawn from this work.

The broad conclusions drawn tentatively from this work are summarized schematically in Fig. 11. Even if the physiological relevance of the endoglucanase/cellulase activity, which has been demonstrated above, turns out to be wrong as a postulate, we would like to point out that in cases in which catalytic, substrate, and product ‘promiscuity’ are all involved, and where the promiscuity involves an enzyme additionally catalyzing a totally different reaction upon a totally different substrate, physiological relevance is not demanded 19, 20. Also, as has been recently pointed out, such promiscuity is often associated with levels of activity, which are orders of magnitude lower than those of any normal enzyme performing the activity 21.

Generally, enzymes from the lower half of the glycolytic pathway (the trunk pathway), such as triosephosphate isomerase (TIM, also known as TPI) and phosphoglycerate kinase, tend to be highly conserved across species. However, all glycolytic enzymes are not conserved to the same level. Differences are known to exist between the EMP pathways of *P. furiosus* and other organisms. Whenever a pathway differs subtly, certain new or modified functions can arise in existing enzymes to potentially compensate for a lost, or altered, function. Changes arise through strategies like gene duplication, sequence divergence, natural selection, and fusions of sections of genes encoding different structural motifs/domains. These result in altered, new, or promiscuous activities; indeed, much evidence exists in the proteomes of organisms to support the likelihood of various such processes having occurred during the evolution of multidomain proteins 12, 22, 23, 24. It is thus conceivable that PfuTIM has evolved, and retained, a promiscuous endoglucanase/cellulase activity that arose at some point during its evolution inside its host organism.

## Materials and methods

### Gene cloning, protein expression, and purification

The gene encoding PfuTIM was originally amplified from the genomic DNA of *P. furiosus* and cloned between the *Bam*H1 and *Hin*dIII sites of vector pQE30 (Qiagen), transformed into XL‐1 Blue *E. coli* cells, as well as into *E. coli* strain M15[pREP4] (Qiagen) for expression. The PCR product was amplified using the following primers: forward—5′‐GCAATCGGATCCATGGCTAAACTCAAGG‐3′ (forward primer with *Bam*H1 site underlined); reverse—5′‐GCAATCAAGCTTCTACTCCTTAATTATTCC‐3′ (reverse primer with *Hin*dIII site underlined). The PCR amplicon was reamplified using Deep Vent DNA polymerase (NEB) and the conditions used were the following: (a) initial denaturation at 95 °C for 5 min followed by (b) 30 cycles each of template denaturation at 98 °C for 1 min, followed by (c) annealing of primers at 52 °C for 2 min and extension at 72 °C for 2 min, followed by (d) a final extension for 10 min at 72 °C. The amplified product and the vector were digested with both *Bam*H1 and *Hin*dIII enzymes and digested vector and insert were ligated using a Quick ligation Kit (NEB) by incubating the reaction at 25 °C for 20–25 min. The ligation mix was then transformed into XL‐1 Blue cells for amplifying and obtaining the plasmid for sequencing and subsequent transformation into M15[pREP4] (Qiagen) cells for expression. Please note that whereas this clone was generated afresh from *P. furiosus* genomic DNA, we have also previously generated and used other clones encoding the same protein, using similar strategies^15^.

A single colony was picked and inoculated into fresh LB medium (10 mL). The primary culture medium was supplemented with antibiotics kanamycin (25 μg·mL^−1^) and ampicillin (100 μg·mL^−1^). The culture was grown overnight at 37 °C with constant shaking at 200 rpm, and addition of 1% of primary inoculum into fresh LB medium on the next day. The culture was then grown for 3–4 h until an O.D. of 0.6 was obtained, after which induction of expression was done with 1 mm isopropylthiogalactoside (IPTG, Calbiochem). The induced culture was grown for a further 4 h at 37 °C, and cells were harvested and purified as per standard Qiagen protocols for purification of expressed protein under native conditions. Affinity purification using Ni‐NTA resin was done at 4 °C, and 1 mm DTT was maintained throughout the procedure. The elution fractions were initially dialyzed against 20 mm Tris, pH 8.0, prior to further purification by gel filtration chromatography using a HiPrep 16/60 Sephacryl S‐200 High Resolution 120 mL column, using either Bio‐Rad's Duo‐Flow chromatographic system or GE's AKTA purifier.

### DNSA assay for endoglucanase/cellulase activity

Endoglucanase activity was checked by the DNSA stopping assay. For this, 40 μm protein (PfuTIM) was taken in 50 mm tris buffer of pH 8.0 containing 100 mm NaCl, and the volume was made up to 1 mL, following which 1 mL of 2% CM‐cellulose solution was added. The tube was incubated at different temperatures ranging from 20 °C °C to 100 °C for 12 h. Then, 3 mL of DNSA reagent was added to each tube. The tubes were boiled for half an hour. The development of brown color resulting from the reaction of the DNSA reagent with reducing sugars in the reaction was measured at 540 nm. Similarly, the pH vs activity profile for the protein was constructed by performing activity measurements in the range of pH from 2 to 10.

### Congo Red plate assay for endoglucanase/cellulase activity

This assay was used as an additional means of visual confirmation of the presence of cellulase activity in a sample of PfuTIM. For this, wells were created in a plate of CM‐cellulose agar (1% CM‐cellulose and 1.5% agar). Protein solutions were placed in these wells and allowed to diffuse into the substrate containing agar on the plates. The plates were incubated overnight at 37 °C to allow the enzyme to act on the substrate. Plates were then stained with 0.1% (w/v) Congo Red for half an hour, followed by washing with 1 m NaCl. The zone of clearance thus obtained is an indicator of the amount of substrate hydrolyzed by the enzyme.

### Cellobioside–resorufin synthetic substrate‐based assay for endoglucanase/cellulase activity

This confirmatory assay was used to calculate various kinetic parameters associated with enzyme activity. For this, a Fluorescent Cellulase Assay Kit (MarkerGene™ Product M1245) was used. The reaction was set up with enzyme concentration of 2 μm and varying substrate concentrations ranging from 2.5 to 250 μm. Experiments were done in triplicate. Kinetics was monitored at 35 °C for 300 s at pH 6.0 using absorption measurements at a fixed wavelength of 570 nm on a Jasco spectrophotometer (V‐600) with a Peltier attachment. Profiling of the pH dependence of activity was also done using the assays in the same manner.

### Zymogram‐based analysis of endoglucanase/cellulase activity

For zymograms, 12% SDS/PAGE gels were used. Resolving gel sections were prepared by copolymerizing CM‐cellulose (i.e., the substrate) with acrylamide, using standard methods. After completion of electrophoresis, proteins in the gel were ‘renatured’ by removing SDS from the gel by repeated soaking in batches of 25% isopropanol, with the assumption being that some fraction of the renatured protein would display enzymatic activity, which could then be monitored visually. Then, the gel was washed in 0.1 m acetate buffer of pH 5.5. The gel was incubated overnight in the same buffer at 4 °C, and subsequently incubated at 37 °C for 1 h before being allowed to be stained by 0.1% Congo Red for half an hour. Thereafter, the gel was washed thoroughly with 1 m NaCl and placed in 0.5% acetic acid. As Congo Red turns blue at low pH, bands (zones of clearance) corresponding to protein displaying hydrolyzing activity against cellulose can be clearly visualized as white or discolored bands against a blue background.

### Difference absorption studies of norharman (inhibitor) binding to PfuTIM

Differential absorption studies were carried out on a Cary 50‐Bio UV–visible absorption spectrophotometer, using a tandem quartz cuvette with two compartments of equal volume and path length. In one compartment, 1.5 mL of beta‐carboline, or norharman, of 300 μm concentration was taken. In the other compartment, 1.5 mL of PfuTIM of 100 μm concentration was taken. The absorption due to the two tandem placed solutions in the cuvette was recorded between 200 and 600 nm, and this absorption was ‘zeroed’ for the entire wavelength range. Then, the cuvette was stoppered, inverted, shaken, and turned upright again, allowing the two solutions to mix and fall back into the two compartments, resulting in an effective halving of the concentrations of each solution and a doubling of the path length of each solution. The absorption due to the solutions in the cuvette was recorded again, and nonzero absorption bands were interpreted in terms of protein–inhibitor interactions.

### Fluorescence studies of norharman (inhibitor) binding to PfuTIM

Tryptophan fluorescence changes were used to study binding of substrate or inhibitor, on a Cary eclipse spectrofluorimeter at 35 °C. The binding of the substrate, or inhibitor, to the enzyme resulted in a decrease in its fluorescence emission intensity, and the proportionality of this decrease to the concentration of substrate, or inhibitor, was measured by monitoring fluorescence emission at 340 nm, using 295 nm excitation. The inhibitor tested was beta‐carboline, or norharman, whereas the substrate tested was cellobioside–resorufin. The concentration range of inhibitor used was 0–200 μm, and the concentration range of substrate used was 0–8 μm.

### Colorimetric endoglucanase activity assay of norharman (inhibitor) influence on PfuTIM

Using a PfuTIM concentration of 2 μm, a norharman (beta‐carboline) concentration of 25 μm and a cellobioside–resorufin (substrate) concentration of 100 μm, we carried out an examination of the effect of the presence of norharman on PfuTIM's endoglucanase activity, measuring color development at 570 nm to monitor activity.

### Studies of the influence of 3‐phosphopropanoic acid (inhibitor, 3‐PPA) on PfuTIM's endoglucanase/cellulase activity

Using a PfuTIM concentration of 2 μm, and various concentrations of 3‐PPA up to 5 mm, as well as a cellobioside–resorufin (substrate) concentration of 100 μm, we carried out an examination of the effect of the presence of 3‐PPA on PfuTIM's endoglucanase activity, measuring color development at 570 nm to monitor activity.

### Native PAGE and Ferguson plot

Native gel high molecular weight (HMW) markers from GE Biosciences were used along with protein samples on native acrylamide gels with five different percentages of acrylamide (5, 7.5, 10, 12.5, and 15%). The relative mobility of each marker was plotted as a function of acrylamide percentage. Negative slopes were calculated from these plots and plotted against the log molecular weight. Data points were fitted to a straight line, and the fit thus obtained was used to determine molecular weights of samples through interpolation.

### Isothermal titration calorimetric (ITC) examination of cellulose binding to PfuTIM

An ITC‐200 instrument (Microcal Inc.) was used to perform isothermal titration calorimetric (ITC) experiments to examine the binding of cellulose to PfuTIM, made possible by the slow activity of the enzyme with the carboxymethyl (CM) cellulosic substrate. For the ITC experiments, conducted at 20 °C, CM‐cellulose was taken in the syringe (144 μm) and PfuTIM was taken in the reservoir (40 μm), and 2 μL of substrate was added repeatedly to a solution of PfuTIM (initially 200 μL), after a first injection of 0.4 μL. Data fitting was done using the origin software provided by Microcal Inc.

### Mutant generation and activity characterization

The following mutants were generated for residues responsible for PfuTIM's activity as TIM (identified through comparison with homologous enzymes): single mutants: (a) Glu141Gly, (b) Lys114Gly, and (c) His96Gly; double mutants: (d) Glu141Gly and Lys114Gly, (e) Lys114Gly and His96Gly, and (f) Glu141Gly and His96Gly; and triple mutant: (g) Glu141Gly, Lys114Gly, and His96Gly. All mutants were cloned in the pQE30 vector between *Bam*H1 and *Hin*dIII sites, like the wild‐type protein. The cloning host used was XL1‐Blue and the expression host used was M15[pREP4]. Mutant proteins were purified under native conditions in the same manner as wild‐type protein. Primers used for inserting mutations are listed (Table 2), in terms of the forward (F) and reverse (R) primers used to insert each mutation. All mutants were made by the standard technique of splicing by overlap extension PCR (SOE‐PCR).

**Table 2:** List of primers for making deficient mutants

	Name of primer	Sequence (5′–3′)
1	PfuTIM K14G Fwd	5′ GCAATCAATTTTGGGACGTACATAGAG 3′
2	PfuTIM K14G –Rev	5′ CTCTATGTACGTCCCAAAATTGATTGC 3′
3	PfuTIM H96G–Fwd	5′CTCCTAAATGGCTCTGAGAATAG 3′
4	PfuTIM H96G –Rev	5′ CTATTCTCAGAGCCATTTAGGAG 3′
5	PfuTIM E144G Fwd	5′ GGTGCTGTAGGGCCTCCTGAGTTG 3′
6	PfuTIM E144G Rev	5′ CAACTCAGGAGGCCCTACAGCAAC 3′

Each mutation site was first individually generated. Double and triple mutants were also generated using the same sets of primers, using overlaps between the primers and the gene‐boundary forward and reverse primers for the wild‐type sequence of the PfuTIM‐encoding gene (described earlier in the section above on cloning of the PfuTIM gene).

## Author contributions

PS made the initial discovery in PG's laboratory, which led to this work. PS and PG together planned all further experiments to verify and understand the observations, analyzed the data and its implications, and together wrote up the manuscript. Experiments were performed by PS under the supervision and guidance of PG.
